# A novel platform using homobifunctional hydrazide for enrichment and isolation of urinary circulating RNAs

**DOI:** 10.1002/btm2.10348

**Published:** 2022-06-03

**Authors:** Bonhan Koo, Yunlim Kim, Yoon Ok Jang, Huifang Liu, Myoung Gyu Kim, Hyo Joo Lee, Myung Kyun Woo, Choung‐Soo Kim, Yong Shin

**Affiliations:** ^1^ Department of Biotechnology, College of Life Science and Biotechnology Yonsei University Seodaemun‐gu, Seoul Republic of Korea; ^2^ Department of Urology, Asan Medical Center University of Ulsan College of Medicine Songpa‐gu, Seoul Republic of Korea; ^3^ Department of Biomedical Engineering School of Electrical Engineering, University of Ulsan Nam‐gu, Ulsan Republic of Korea; ^4^ Department of Urology Ewha Womans University Mokdong Hospital Yangcheon‐gu, Seoul Republic of Korea

**Keywords:** benign prostatic hyperplasia, circulating RNA, enrichment, homobifunctional hydrazide, prostate cancer

## Abstract

Changes in specific circulating RNA (circRNA) expressions can serve as diagnostic noninvasive biomarkers for prostate cancer (PCa). However, there are still unmet needs, such as unclear types and roles of circRNAs, PCa detection in benign prostatic hyperplasia (BPH) by unstandardized methods, and limitations of sample volume capacity and low circRNA concentrations. This study reports a simple and rapid circRNA enrichment and isolation technique named “HAZIS‐CirR” for the analysis of urinary circRNAs. The method utilizes homobifunctional hydrazides with amine‐modified zeolite and polyvinylidene fluoride (PVDF) syringe filtration for combining electrostatic and covalent coupling and size‐based filtration, and it offers instrument‐free isolation of circRNAs in 20 min without volume limitation, thermoregulation, and lysis. HAZIS‐CirR has high capture efficiency (82.03%–92.38%) and a 10‐fold more sensitive detection limit (20 fM) than before enrichment (200 fM). The clinical utility of HAZIS‐CirR is confirmed by analyzing circulating mRNAs and circulating miRNAs in 89 urine samples. Furthermore, three miRNA panels that differentiate PCa from BPH and control, PCa from control, and BPH from control, respectively, are established by comparing miRNA levels. HAZIS‐CirR will be used as an optimal and established method for the enrichment and isolation of circRNAs as diagnostic, prognostic, and predictive biomarkers in human cancers.

## 1 INTRODUCTION

Circulating nucleic acids (NAs), first described in 1948, are stable circulating DNA and RNA molecules in body fluids, such as plasma, urine, and saliva, and are considered promising diagnostic, prognostic, and predictive noninvasive biomarkers.^1^, ^2^ These molecules originate from necrotic and apoptotic cells and are secreted by a variety of cells to communicate with other cells, even at the early stages of cancer.^3^, ^4^, ^5^ Changes in the expression of these circulating NAs directly influence physiology and pathology, such as cancer development and progression.^6^, ^7^ Circulating NAs are used as cancer biomarkers in liquid biopsy without the disadvantages of conventional biopsy techniques, such as invasiveness, high risk, high cost, and tumor location and size limitations.^8^, ^9^ In addition, because of the heterogeneity of circulating NAs by type and stage of cancer, it is possible to monitor cancer metastasis and cancer treatment prognosis through molecular characterization.^10^, ^11^ Due to these advantages, circulating NAs are used as biomarkers for early or accurate diagnosis of cancer, and the diagnostic platform used to detect them should be highly sensitive, such as end‐point, real‐time, and droplet‐digital PCR. However, the low sensitivity due to low concentrations of circulating NAs in liquid specimens is one challenge to consider in the development of a powerful diagnostic platform. Currently, there is a lack of discussion about the enrichment of high‐concentration circulating NAs using a large sample volume.

Prostate cancer (PCa) is one of the most common malignancies in men worldwide and the second leading cause of all cancer‐related deaths.^12^, ^13^ Prostate‐specific antigen (PSA) testing has been widely used for the early diagnosis of PCa, consequently increasing the number of treatable patients with PCa and dramatically reducing the death rate from PCa.^14^ PSA is only present in prostate tissue, and PSA levels are elevated in the serum of patients with PCa.^15^ However, PSA levels are variously increased by many factors.^16^ Especially, benign prostatic hyperplasia (BPH) is the most common condition associated with elevated PSA values that can be mistaken for PCa. In the PSA gray zone of 4–10 ng ml^−1^, some limitations were closely related to certain nonmalignant conditions, such as BPH and prostatitis.^17^, ^18^, ^19^ The low sensitivity and specificity of the PSA test lead to erroneous diagnosis and treatment, because PCa patients sometimes have positive BPH findings with or without clinical symptoms. Erroneous diagnosis can cause a lot of stress for patients and put a lot of burden on the healthcare system.^20^, ^21^ Therefore, there is an unmet need for more effective biomarkers for PCa diagnosis. Recently, due to the various advantages of circulating NAs, PCa diagnostic platforms for circulating RNAs (circRNAs), including circulating miRNA and circulating mRNA, are being intensively developed.^22^, ^23^, ^24^ miRNA is a noncoding RNA molecule approximately 22 nucleotides long (17–27 nts) that preferentially binds to the 3′UTR of mRNA and acts as a post‐transcriptional regulator, influencing important cellular processes, such as cell cycle, proliferation, and apoptosis.^25^, ^26^ In patients with PCa, circulating *miRNA‐141‐3p* and *miRNA‐375‐3p* are increased compared to control patients and are known as promising biomarkers.^22^, ^27^, ^28^ However, the role of these miRNAs is still unclear, and there are limitations to the discovery of PCa‐specific miRNA biomarkers among numerous miRNAs. Furthermore, previous research has mostly focused on circulating miRNA rather than circulating mRNA.

One of the important considerations for circRNA analysis is the sample extraction method to obtain high‐concentration circRNAs.^29^, ^30^, ^31^, ^32^ To capture rare circRNAs, conventional isolation and purification technologies using phenol‐chloroform‐based, spin‐column‐based, or bead‐based methods have been proposed.^33^, ^34^, ^35^ These approaches are expensive, time‐consuming (>30 min), labor‐intensive, depend on hazardous chemicals, such as phenol, and require large or specialized equipment for centrifugation and temperature control. In addition, they use a lysis buffer containing a chaotropic reagent, such as guanidine thiocyanate, which increases the genetic background derived from noncancerous cells. It is also impossible to process a large volume of clinical specimens, so the reproducibility is poor due to the use of low concentrations of circRNAs. Therefore, developing a universal technology for the enrichment and isolation of circRNAs with high reproducibility remains a challenge.

Here, we developed a novel platform called “HAZIS‐CirR” for the simple and rapid enrichment and isolation of circRNAs from the urine of patients with PCa, BPH, and control patients by combining adipic acid dihydrazide (ADH), amine‐modified zeolite (AZ), and PVDF syringe filtration. ADH is low‐cost, water‐soluble, and odorless with low toxicity. As a nucleophilic molecule, ADH binds covalently to amine groups. It is mainly used as a crosslinking agent in the manufacture of mechanical latex films and injectable oxidized hyaluronic acid hydrogels due to its reactive hydrazide group at each end of the molecule.^36^, ^37^, ^38^ Zeolites are natural mesoporous and microporous aluminosilicates widely used in various industrial processes, such as separation, catalysis, and sensors because of their low‐cost, structural stability, and biocompatibility advantages.^39^, ^40^, ^41^, ^42^ The large surface area relative to the volume of zeolite increases its reactivity with various biomaterials, so zeolite is easily manufactured into functionalized materials for the binding of biomolecules through surface modification. We exploited these properties of ADH to couple AZ and circRNAs through covalent and electrostatic interaction. Furthermore, we used PVDF syringe filters, which can handle large samples and large volumes easily and simply without relying on large equipment, such as centrifuge and vacuum pump. The PVDF syringe filter requires only a suitable syringe for the sample volume, can quickly separate substances larger than the filter pore size without external contamination, and allows washing debris smaller than the pore size of the filter.

We analyzed several circulating mRNAs and circulating miRNAs using a total of 89 urine samples in HAZIS‐CirR. Our results demonstrated that circRNAs can be concentrated and extracted quickly, inexpensively, and effectively using urine from patients with PCa (55 samples), BPH (24 samples), and control patients (10 samples), and circulating mRNAs and circulating miRNAs that can be used as PCa biomarkers in patients with BPH. Furthermore, we identified three miRNA panels that distinguish PCa from BPH and control samples (Panel 1), PCa from control samples (Panel 2), and BPH from control samples (Panel 3), respectively, by comparing the miRNA levels. This platform provides a technique for enrichment and isolation of circRNAs, which are secreted to tumor sites and found in various body fluids, and for high‐concentration biomarker analysis of PCa, including early‐stage PCa.

## 2 RESULTS

### 2.1 Enrichment and isolation of circRNAs using HAZIS‐CirR

HAZIS‐CirR is a novel platform for the enrichment and isolation of circRNAs based on the molecular characteristics of AZ and ADH and the size‐based filtering of PVDF sterilized filters. As a porous structure and amine group donor, AZ can effectively capture circRNAs due to the electrostatic coupling between the positively charged amino group and the negatively charged phosphate of the circRNAs and can also capture ADH and circRNAs due to the covalent coupling. ADH is a pH‐sensitive linker and a nonchaotropic reagent, with a C4 backbone and two reactive hydrazide groups (C=ONHNH_2_). The hydrazide group of ADH can react with carbonyl groups of circRNAs to form a reactive hydrazone bond. In addition, because it has two nucleophilic primary amine groups and one aldehyde group per hydrazide group, it can react with amine‐reactive and aldehyde‐reactive groups of AZ and circRNAs. The PVDF syringe filter can process samples without limitation of sample volume and allows simple, quick, and user‐friendly sample preparation. The combination of these three materials—AZ, ADH, and PVDF syringe filter—enables simple and rapid enrichment and isolation of circRNAs.

**FIGURE 1:**
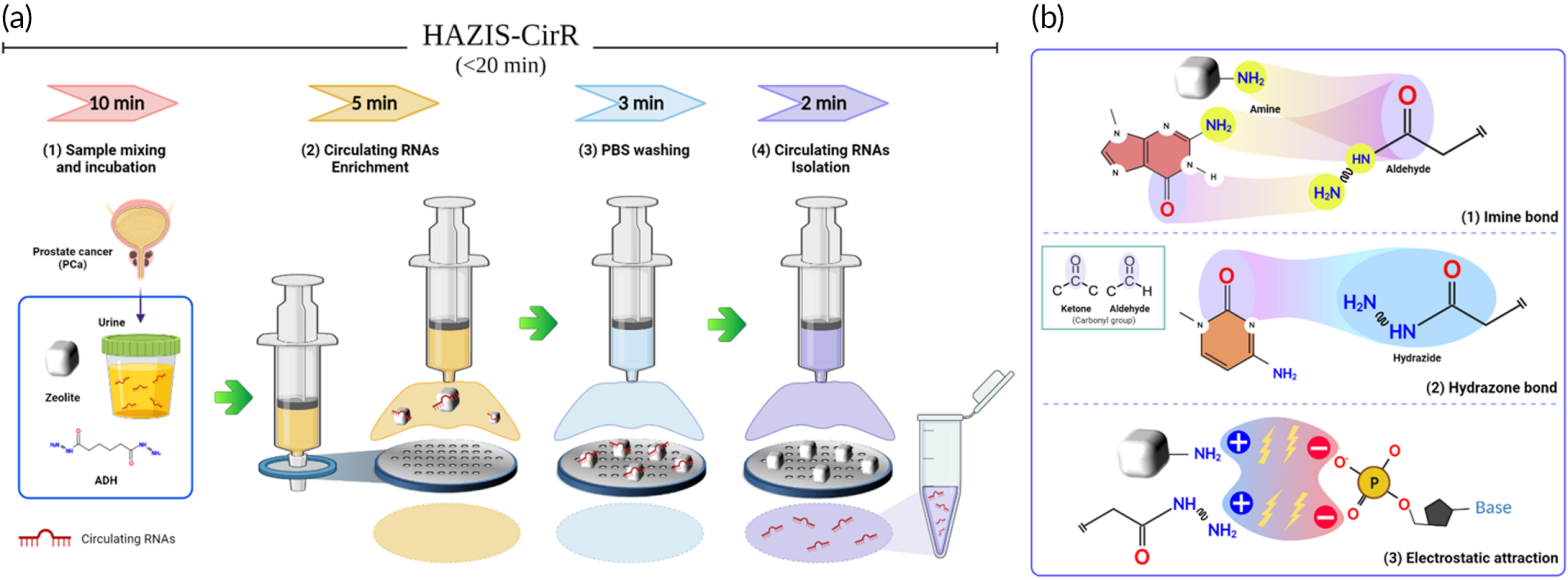
Schematic presentation of HAZIS‐CirR. (a) Schematic process of HAZIS‐CirR for simple and rapid enrichment and extraction of circulating RNAs from clinical specimens. (1) Sample mixing and incubation: AZ and ADH are added to the urine sample, and during incubation for 10 min, circulating RNAs, AZ, and ADH interact via covalent and electrostatic coupling. (2) Enrichment of circulating RNAs: a urine sample is injected through the PVDF syringe filter. Circulating RNAs attached to the surface of AZ are filtered by the pore size of the syringe filter, designed to remove debris and nonreactive biomaterials. (3) Washing: remaining contaminants are removed by washing with PBS. (4) Isolation of circulating RNAs: the pH (10.6) based elution buffer hydrolyzes the electrostatic and covalent bonds and passes through a filter to isolate circulating RNAs. Circulating RNAs were concentrated and extracted within 20 min using HAZIS‐CirR and stored at sub‐zero temperature (−20 or −80°C) until further use. (B) Mechanism of covalent and electrostatic coupling in HAZIS‐CirR. (1) Imine bond: reaction of amines with carbonyls. (2) Hydrazone bond: reaction of hydrazides with carbonyls. (3) Electrostatic attraction: reaction of positively charged amines with negatively charged phosphate moieties. This image was created using BioRender (https://biorender.com). AZ, amine‐modified zeolite; ADH, adipic acid dihydrazide; PVDF, polyvinylidene fluoride

Figure 1a illustrates HAZIS‐CirR designed for enrichment and isolation of circRNAs from urine samples of either PCa or BPH or control patients. The process of HAZIS‐CirR for enrichment and isolation of circRNAs consists of four steps: (1) sample mixing and incubation, followed by (2) enrichment, (3) washing, and (4) isolation of circRNAs. These procedures do not require any additional steps before operation other than the functionalization of zeolite and direct interaction between AZ, ADH, and circRNAs. The process can be completed within 20 min, including a 10‐min incubation. The covalent and electrostatic coupling between AZ, ADH, and circRNAs in the sample mixing and incubation step is presented in Figure 1b. The binding mechanisms can be explained as follows: (1) the aldehyde group of ADH reacts with the amine groups of AZ, and the nitrogenous base (G, A, and C) of the RNA nucleotide and the amine group of ADH reacts with the carbonyl groups of the nitrogenous base (G, C, and U) to form a reactive imine bond. (2) The hydrazide group of ADH reacts with the carbonyl groups of the nitrogenous base (G, C, and U) of the RNA nucleotide to form a reactive hydrazone bond. (3) The positive charge of AZ and ADH, respectively, reacts with the negative charge of the phosphate group of the RNA nucleotide by electrostatic attraction. Functional groups of the nitrogenous base are present in Figure S1. circRNAs captured on the AZ surface are injected into a PVDF syringe filter. Debris and unbound molecules smaller than the filter pore size (0.45 μm) pass through the filter and are discarded, and circRNAs are concentrated on the filter surface by AZ. Residual debris is removed by washing with PBS, and circRNAs are extracted by alkaline hydrolysis of the reactive bonds between AZ, ADH, and circRNAs using elution buffer (pH 10.6). Without a cell lysis step, there is no contamination by cellular background NAs that occurs during cell lysis. Moreover, our procedure uses a nonchaotropic agent to avoid degradation of the extracted RNAs. In addition, high‐concentration and high‐purity RNAs can be extracted quickly and simply without electricity and specialized equipment, such as centrifuge, vacuum pump, and thermoregulator, used in conventional methods.

### 2.2 Characterization of HAZIS‐CirR

**FIGURE 2:**
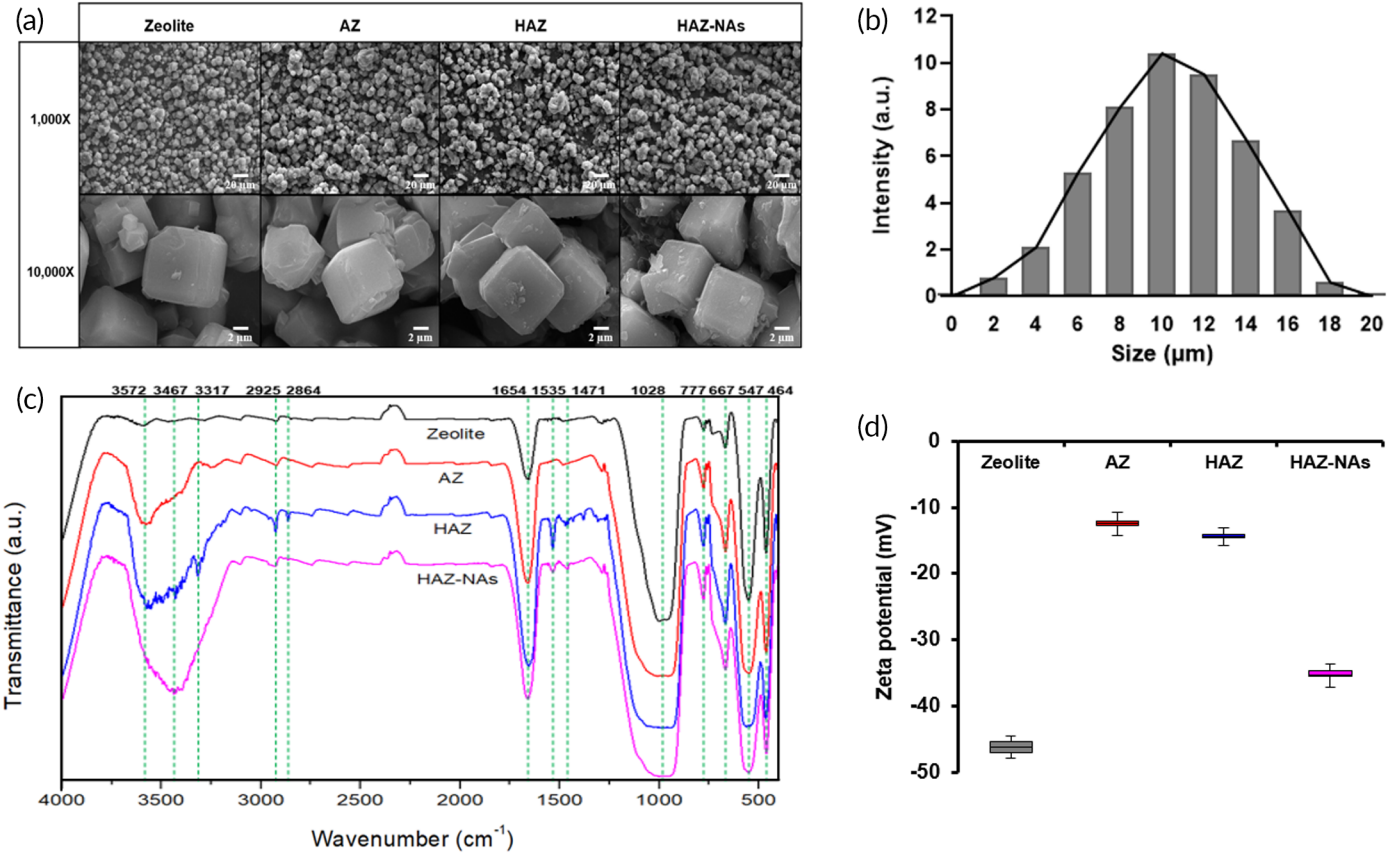
Characterization of pure zeolite and modified zeolites (AZ, HAZ, and HAZ‐NAs). (a) SEM images of pure and modified zeolites at 1000× and 10,000× magnifications. (b) Size distribution of zeolite. (c) FTIR spectra of pure zeolite and modified zeolites. The green dotted line indicates the wavenumber of the major characteristic peaks. (d) Zeta‐potential of pure zeolite and modified zeolites. Error bars indicate standard deviation of the mean of at least three independent experiments. AZ, amine‐modified zeolite; HAZ, hydrazide‐modified AZ; HAZ‐NAs, HAZ with nucleic acids; SEM, scanning electron microscope; FTIR, Fourier‐transform infrared spectroscopy

We first characterized HAZIS‐CirR to identify the molecular properties and binding mechanisms between AZ, ADH, and NAs. The shape and size distribution of zeolite were confirmed from scanning electron microscope (SEM) images and dynamic light scattering (DLS) analysis (Figure 2a,b). Zeolite is about 2–18 μm (mostly 6–14 μm) and maintains a stable structure after silanization and even after interaction with ADH and NAs. Fourier‐transform infrared spectroscopy (FTIR) results (Figure 2c) for pure zeolite showed absorption peaks at 464 cm^−1^ (symmetric bending of TO, T: tetrahedrally bonded Si or Al), 547 cm^−1^ (double six‐membered rings of TOT symmetric stretching), 667 cm^−1^ (SiOSi symmetric stretching), 777 cm^−1^ (symmetric TOT stretching), 1028 cm^−1^ (SiOAl asymmetric stretching of TO), and 1654 cm^−1^ (OH bending) and broad absorption bands at 3467–3572 cm^−1^ (hydroxyl group). After silanization, AZ showed an increase in the intensity of the absorption peaks at 1654 cm^−1^ and the broad absorption bands at 3467–3572 cm^−1^ due to NH stretching and NH bending, respectively. These results verified that amine groups are formed on the AZ surface. In addition, the ADH‐modified AZ (HAZ) showed additional absorption peaks at 1471 cm^−1^ (CN stretching), 1535 cm^−1^ (NH bending), 2864 cm^−1^ (CH stretching and bending), 2925 cm^−1^ (CH stretching), and 3317 cm^−1^ (NH stretching), increased intensity of the absorption peak at 1654 cm^−1^ (C=O stretching) and increased intensity of the broad absorption bands at 3467–3572 cm^−1^ (NH stretching and bending) due to ADH (Figure S2A). These FTIR results provided spectroscopic evidence that AZ and ADH can interact to form an imine bond (Figure 1b) and that hydrazide is generated on the surface of AZ, which can be used as a functional group. Furthermore, the FTIR data for HAZ with NAs (HAZ‐NAs) showed that absorption peaks representing hydrazide (1471, 1535, 2864, 2925, and 3317 cm^−1^) are decreased by the imine bond, hydrazine bond, and electrostatic attraction, and the broad absorption peaks (3317–3572 cm^−1^) caused by NAs are increased. On the basis of the FTIR data, AZ and ADH can be used to capture circRNAs. Figure 2d shows the zeta‐potential of pure zeolite, AZ, HAZ, and HAZ‐NAs. Pure zeolite showed a zeta‐potential of −44.58 mV attributed to the OH and oxygen‐containing groups on the zeolite surface. For AZ, the zeta‐potential increased to −11.62 mV compared to pure zeolite due to the immobilized amine group on the zeolite surface. The zeta‐potential of −13.82 mV obtained for HAZ is similar to that of AZ. This result is attributed to the interaction between AZ and ADH and the amines and aldehydes of the hydrazide groups. The high zeta‐potential of HAZ‐NAs (−33.92 mV) can be explained by the strong negative charge of the phosphate groups of NAs binding with HAZ. Our zeta‐potential data confirmed that AZ and HAZ have high zeta‐potential values and that electric bonds are formed from the interaction of HAZ with the negative charge of circRNAs. These characterization results indicate that HAZIS‐CirR can be used as a new platform for the enrichment and isolation of circRNAs.

### 2.3 Optimization of HAZIS‐CirR

**FIGURE 3:**
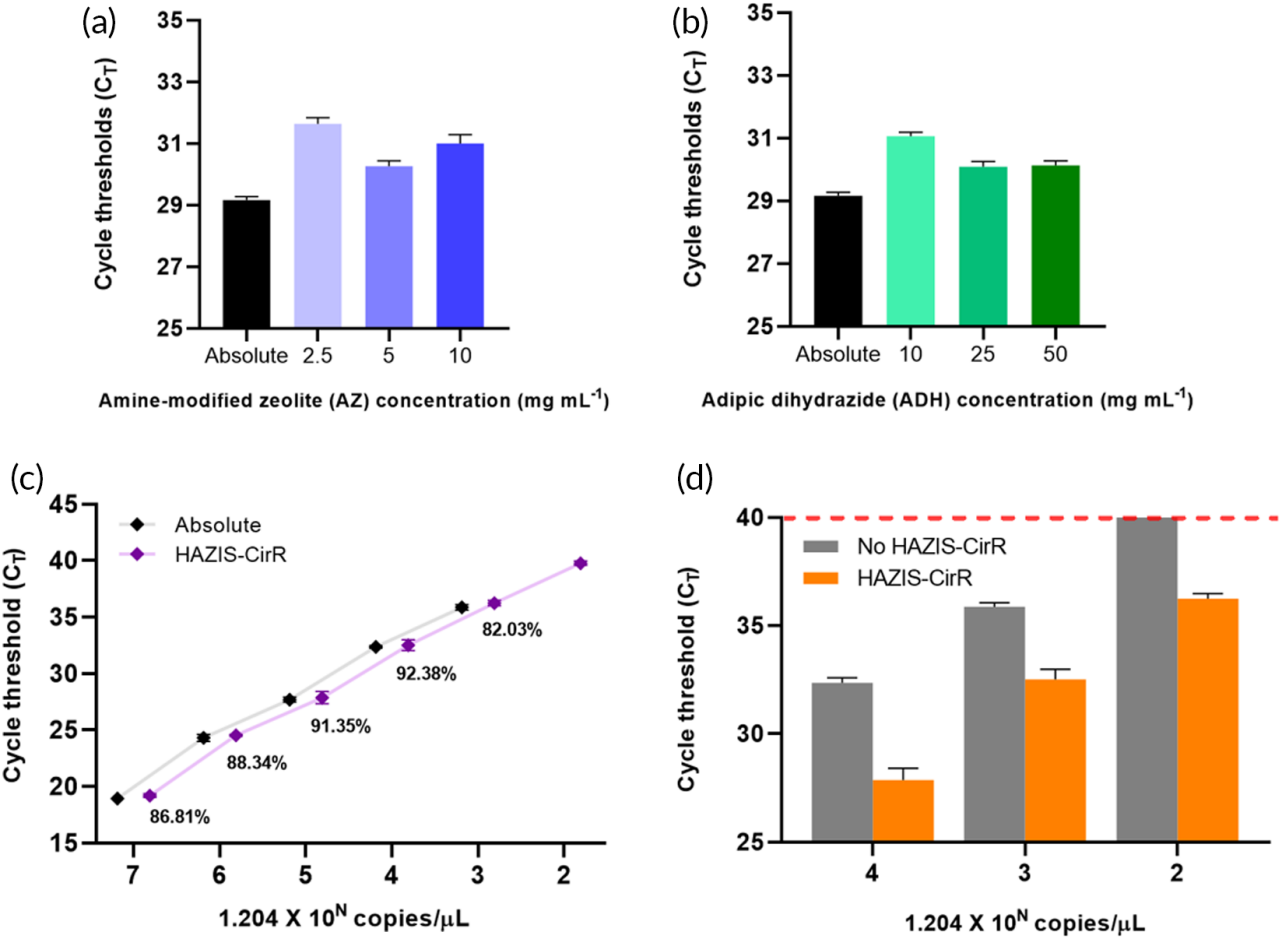
Optimization of HAZIS‐CirR. (a) Comparison of capture efficiency of circulating RNAs according to AZ concentration (2.5, 5.0, and 10 mg ml^−1^) and (b) ADH concentration (10, 25, and 50 mg ml^−1^). (c) Capture efficiency of HAZIS‐CirR according to the concentration of circulating RNAs. Capture efficiency was calculated by quantitative comparison using the linear relationship of the standard curve. (d) Detection limit before and after HAZIS‐CirR. Error bars indicate standard deviation of the mean of at least three independent experiments. AZ, amine‐modified zeolite; ADH, adipic acid dihydrazide

We next determined the optimized concentrations of AZ and ADH in HAZIS‐CirR. Optimization of the AZ and ADH concentrations is essential to avoid the low‐binding affinity of circRNAs due to the low concentration and low capture efficiency of circRNAs because of the interference by molecules of high concentration. The optimization was set to 1 ml of the sample, and AZ and ADH were added at the same ratio according to the change in the sample volume. We varied the concentrations of AZ and ADH, which revealed that the best capture efficiency of RNAs was obtained when 5 mg of AZ and 50 mg of ADH were used per 1 ml of sample (Figure 3a,b). In addition, the capture efficiency was assessed using five homobifunctional hydrazide (HH) candidates—ADH, carbonic dihydrazide (CDH), oxalyl dihydrazide (ODH), malonic dihydrazide (MDH), and succinic dihydrazide (SDH)—in HAZIS‐CirR, among which, ADH presented the best capture efficiency (Figure S2B‐C).

Moreover, we identified the detection limit and capture efficiency of HAZIS‐CirR. The capture efficiency was compared with the copy number before and after HAZIS‐CirR using the same concentration of *hsa‐mir‐21‐5p* ss mimics and T7 in vitro transcribed RNAs of 150 bp and 112 bp containing prostate cancer antigen 3 gene (*PCA3*) and 18S ribosomal RNA (18S rRNA) target genes. It was calculated using the formula value of the standard curve obtained by serial dilution of *hsa‐mir‐21‐5p* ss mimics (Figure S3 and Table S1) and T7 in vitro transcribed RNAs (Figure S4A–C and Table S2). As shown in Figure 3c and Table S3, the detection limit before and after HAZIS‐CirR remained the same (1.204 × 10^3^ copies reaction^−1^), and the enrichment and isolation of circRNAs using *hsa‐mir‐21‐5p* ss mimics could be carried out with high efficiency (82.03%–92.38%). Long RNA fragments (>100 bp) can be concentrated and isolated with high efficiency at both 150 bp (82.14%–95.48%) and 112 bp (80.37%–94.53%) size (Figure S4D,E and Table S4). These results showed that HAZIS‐CirR has a similar capture efficiency at both long RNA fragments (150 bp and 112 bp) and short RNA fragments (*hsa‐mir‐21‐5p* ss mimic, 20 bp) and can isolate circRNAs of various lengths. Furthermore, we compared the capture efficiency of *hsa‐mir‐21‐5p* ss mimics before and after HAZIS‐CirR using a large volume of samples and human serum. When the *hsa‐mir‐21‐5p* ss mimics were concentrated using a large volume (from 1 ml to 100 μl) of sample in HAZIS‐CirR, the sensitivity of the detection limit increased 10‐fold to 20 fM (1.204 × 10^2^ copies reaction^−1^) compared to not proceeding with enrichment (Figure 3d). As shown in Figure S5A and Table S5, it was confirmed that the *hsa‐mir‐21‐5p* ss mimic can be concentrated and isolated with high efficiency in both 5 ml (78.65%–94.51%) and 10 ml (82.49%–98.25%). These results showed that HAZIS‐CirR is useful for various clinical sample volumes (1–10 ml). In addition, the *hsa‐mir‐21‐5p* ss mimic can be concentrated and isolated with high efficiency in the human serum sample (80.62%–96.36%) (Figure S5B and Table S6). These results showed that HAZIS‐CirR is useful for a variety of liquid biopsies including urine, blood plasma. Our optimization data indicate that HAZIS‐CirR can increase the circRNA capture efficiency and sensitivity without the limitations of volume capacity, lengths of RNAs, and various types of liquid samples.

### 2.4 Circulating mRNAs in clinical specimens using HAZIS‐CirR

**FIGURE 4:**
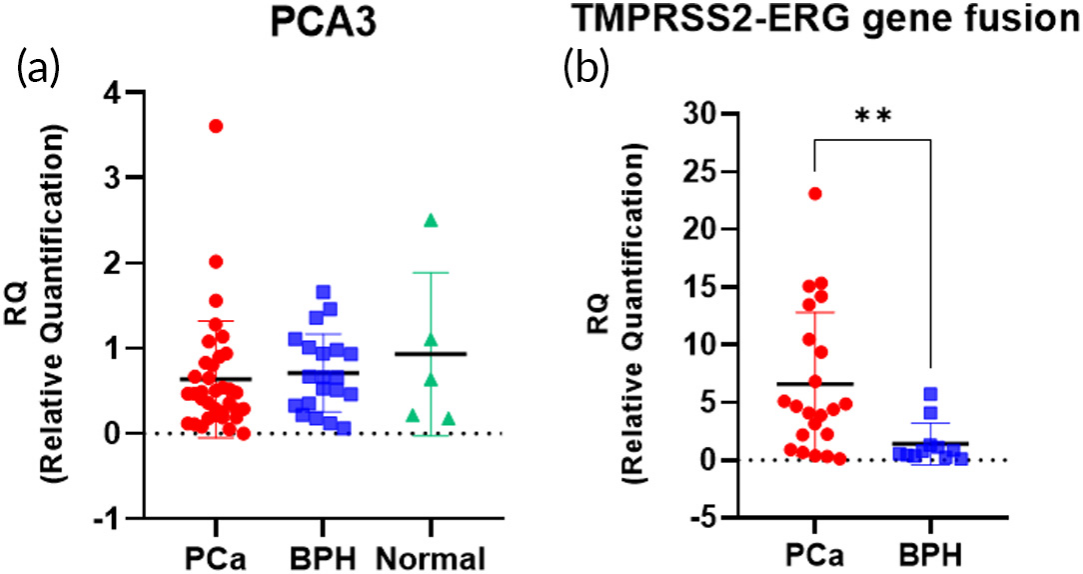
Clinical utility of circulating mRNAs via HAZIS‐CirR. (a,b) Relative quantification (RQ) analysis via HAZIS‐CirR for (a) *PCA3* gene using a total of 60 samples (35 PCa, 20 BPH, and 5 controls) and (b) *TMPRSS2‐ERG* gene fusion using a total of 33 samples (22 PCa and 11 BPH). Significant differences between groups are indicated: *p < 0.05, **p < 0.01, and ***p < 0.001. PCa, prostate cancer; BPH, benign prostatic hyperplasia

We analyzed the circulating mRNAs in 84 urine samples to confirm the clinical utility of HAZIS‐CirR for circulating mRNA‐based PCa diagnosis (Figure S6) using the *PCA3* and the *TMPRSS2‐ERG* gene fusion as the biomarkers and the relative quantification (RQ) values were calculated (Figure 4a,b). The *PCA3* gene is a long noncoding RNA overexpressed in PCa cells, and it has recently been used in clinical applications as a PCa biomarker.^43^, ^44^ The *ERG* oncogene is known to be overexpressed in more than 50% of PCa cases, and *ERG* overexpression is induced by fusion with *TMPRSS2*, a prostate‐specific and androgen‐regulating gene, and *TMPRSS2‐ERG* gene fusion is a mutation found in 40%–70% of PCa cases.^45^, ^46^, ^47^ Recent studies have highlighted the value of this biomarker combination.^48^, ^49^ We found that *PCA3* was not significantly different among the PCa (mean RQ = 0.64 ± 0.68), BPH (mean RQ = 0.71 ± 0.46), and control samples (mean RQ = 0.93 ± 0.96). Conversely, *TMPRSS2‐ERG* was highly overexpressed in the urine of patients with PCa (mean RQ = 6.58 ± 6.23) compared to patients with BPH (mean RQ = 1.41 ± 1.81; *p* < 0.01). It is evident that *TMPRSS2‐ERG*, as a biomarker for PCa, shows a clear difference compared to BPH, but *PCA3* does not show a significant difference among the PCa, BPH, and control samples.

### 2.5 Circulating miRNAs in clinical specimens using HAZIS‐CirR

**FIGURE 5:**
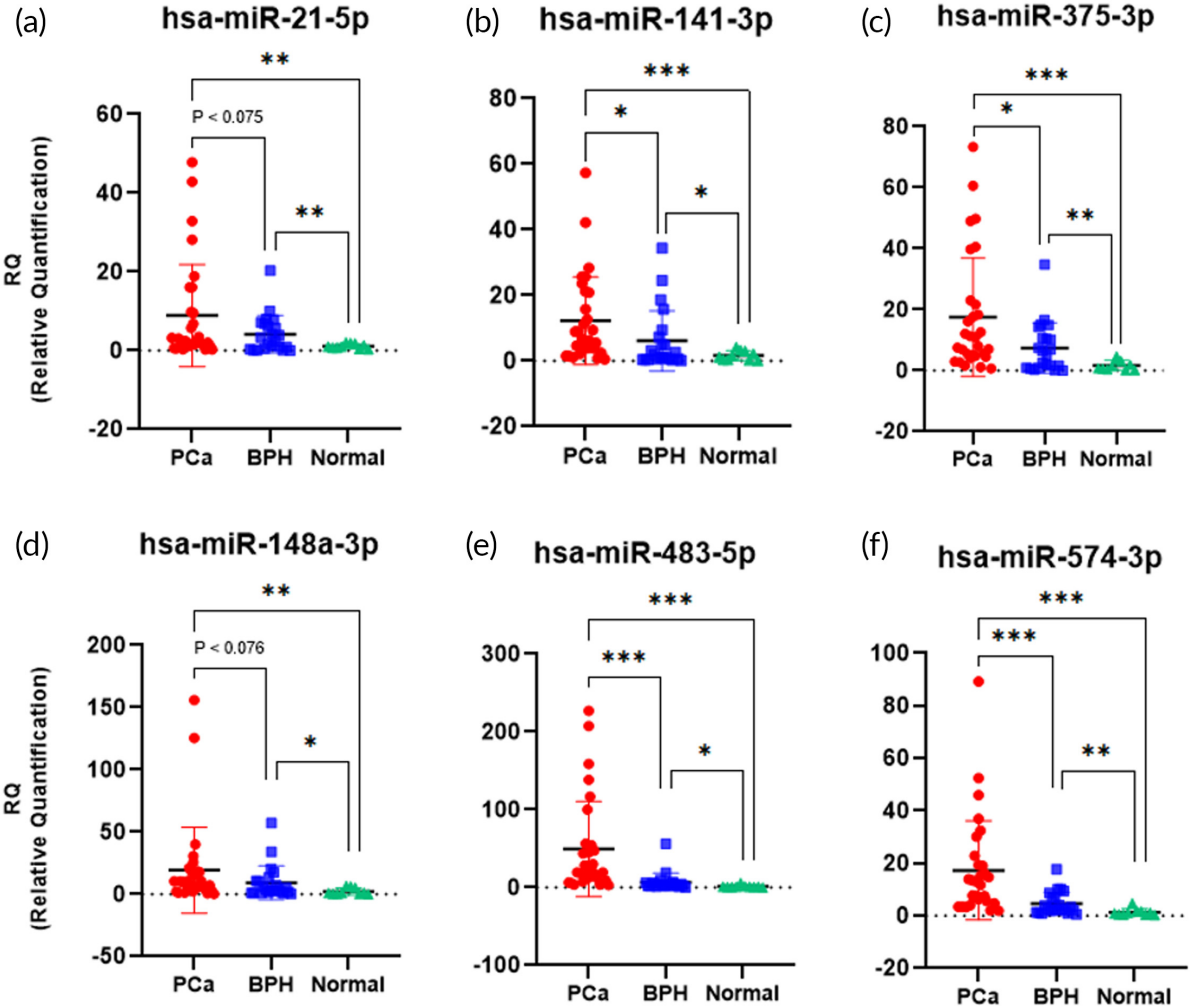
Clinical utility of circulating miRNA via HAZIS‐CirR. (a–f) Relative quantification (RQ) analysis of (a) *hsa‐miR‐21‐5p*, (b) *hsa‐miR‐141‐3p*, (c) *hsa‐miR‐375‐3p*, (d) *hsa‐miR‐148a‐3p*, (e) *hsa‐miR‐483‐5p*, and (f) *hsa‐miR‐574‐3p* using a total of 62 samples (30 PCa, 22 BPH, and 10 controls). Significant differences between groups are indicated: *p < 0.05, **p < 0.01, and ***p < 0.001. PCa, prostate cancer; BPH, benign prostatic hyperplasia

In addition, we also analyzed the circulating miRNAs in 62 urine samples to confirm the clinical utility of HAZIS‐CirR for circulating miRNA‐based PCa diagnosis (Figures 5 and S6). To this end, we used three known PCa biomarkers (*miR‐21‐5p*, *miR‐141‐3p*, and *miR‐375‐3p*) as circulating miRNA biomarkers, and the RQ values were calculated. *MiR‐21‐5p* is an oncogenic microRNA and is known to be overexpressed in several cancers, including PCa.^50^, ^51^ Likewise, *miR‐141‐3p and miR‐375‐3p* have similar expression patterns to *miR‐21‐5p* and are known to be overexpressed in PCa.^22^, ^27^, ^28^ Our results confirmed that *miR‐21‐5p* (mean RQ = 8.91 ± 12.92; *p* < 0.01), *miR‐141‐3p* (mean RQ = 12.26 ± 13.37; *p* < 0.001), and *miR‐375‐3p* (mean RQ = 17.30 ± 18.69; *p* < 0.001) were overexpressed in the urine from patients with PCa compared to the control patients (mean RQ = 1.16 ± 0.62, 1.69 ± 1.44, and 1.73 ± 1.64, respectively), demonstrating that *miR‐21‐5p*, *miR‐141‐3p*, and *miR‐375‐3p* can be used as PCa biomarkers. Even in patients with BPH, *miR‐21‐5p* (mean RQ = 4.22 ± 4.75; *p* < 0.01), *miR‐141‐3p* (mean RQ = 5.04 ± 6.68; *p* < 0.05), and *miR‐375‐3p* (mean RQ = 7.57 ± 8.21; *p* < 0.01) were overexpressed compared to control patients. This suggests that there must be a significant difference in these biomarkers between patients with PCa and BPH. Additionally, *miR‐141‐3p* (*p* < 0.05) and *miR‐375‐3p* (*p* < 0.05) were overexpressed in PCa compared to BPH, and there was no significant difference in *miR‐21‐5p* (*p* < 0.075). As a result, we were able to verify that *miR‐141‐3p and miR‐375‐3p* can be used as biomarkers to discriminate patients with PCa from those with BPH.

Next, we sought to discover potential biomarkers that can differentiate PCa from BPH patients by analyzing the clinical utility of *miR‐148a‐3p*, *miR‐483‐5p*, *and miR‐574‐3p*.^52^, ^53^, ^54^ Three miRNAs were observed to be upregulated in PCa cell lines and tissues in a recent study, and consistent overexpression of these miRNAs was found in several types of clinical samples, including plasma and urine from PCa patients.^20^, ^21^, ^55^ Furthermore, these miRNAs have been implicated in the presence of cancer and have been shown to promote various cancer progression, migration, or invasion.^56^, ^57^, ^58^, ^59^ Our results confirmed that *miR‐148a‐3p* (mean RQ = 19.45 ± 34.51; *p* < 0.01), *miR‐483‐5p* (mean RQ = 49.53 ± 60.83; *p* < 0.001), and *miR‐574‐3p* (mean RQ = 17.28 ± 18.82; *p* < 0.001) were overexpressed in PCa compared to the control samples (mean RQ = 2.27 ± 2.73, 1.44 ± 1.58, and 1.35 ± 1.29, respectively). However, *miR‐148a‐3p* (mean RQ = 9.32 ± 13.51; *p* < 0.05), *miR‐483‐5p* (mean RQ = 6.98 ± 11.64; *p* < 0.05), and *miR‐574‐3p* (mean RQ = 4.68 ± 4.34; *p* < 0.01) were also overexpressed in BPH compared to control samples. When comparing PCa and BPH, we verified that *miR‐483‐5p* (*p* < 0.001) and *miR‐574‐3p* (*p* < 0.001) were overexpressed in the urine of patients with PCa compared to those with BPH, and there was no significant difference in *miR‐148a‐3p* (*p* < 0.076). These results show that *miR‐483‐5p and miR‐574‐3p* are suitable biomarkers that can differentiate between PCa and BPH.

**FIGURE 6:**
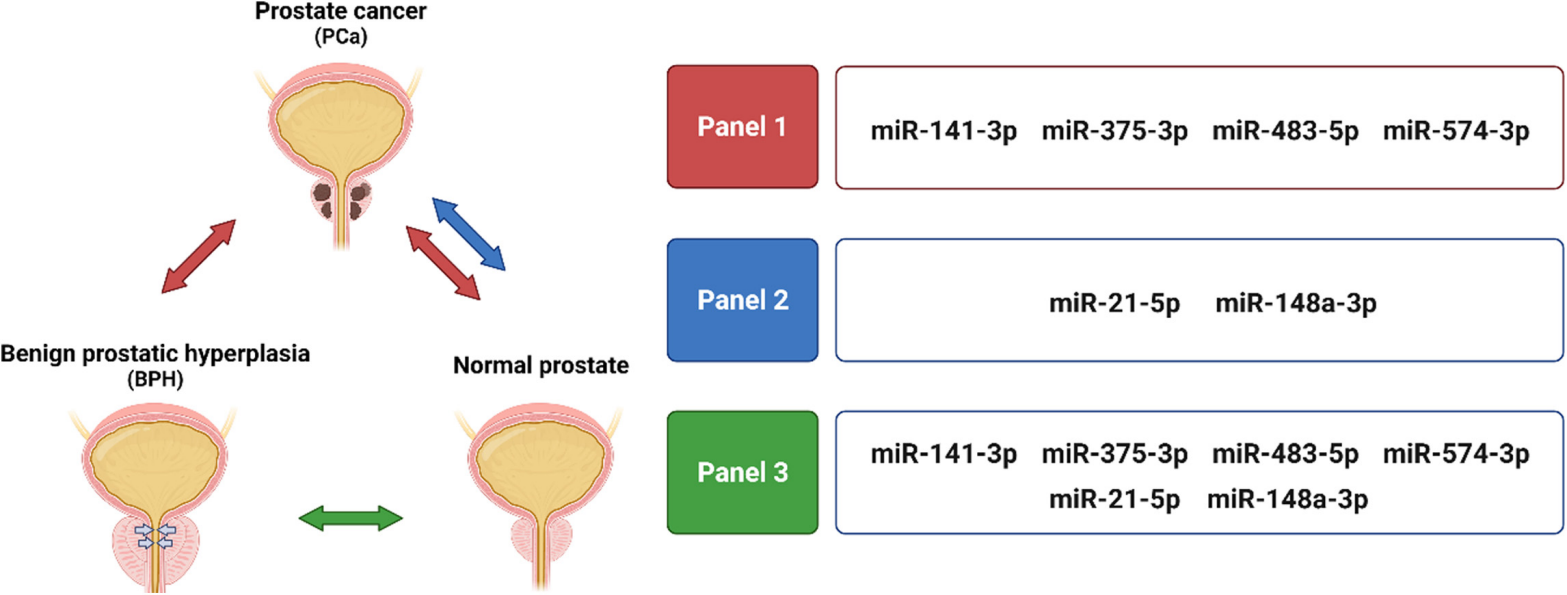
Three miRNAs panels of circulating miRNAs via HAZIS‐CirR. Panel 1 consists of *miR‐141‐3p*, *miR‐375‐3p*, *miR‐483‐5p*, and *miR‐574‐3p* for PCa diagnosis and differentiation from patients with BPH and controls. Panel 2 consists of *miR‐21‐5p* and *miR‐148a‐3p* for PCa diagnosis and differentiation from controls. Panel 3 consists of *miR‐21‐5p*, *miR‐141‐3p*, *miR‐148a‐3p*, *miR‐375‐3p*, *miR‐483‐5p*, and *miR‐574‐3p* for BPH diagnosis and differentiation from controls. This image was created using BioRender (https://biorender.com). PCa, prostate cancer; BPH, benign prostatic hyperplasia

Although *miR‐21‐5p* and *miR‐148a‐3p* show high expression rates in PCa, they are not suitable biomarkers for distinguishing between PCa and BPH because they are not statistically significant compared to BPH. Based on these results, we identified three panels of miRNAs as diagnostic biomarkers for PCa (Figure 6). Panel 1 consists of *miR‐141‐3p*, *miR‐375‐3p*, *miR‐483‐5p*, and *miR‐574‐3p*, which show significant differences in PCa compared to BPH and control samples. All miRNAs in Panel 1 were statistically significant (*p* < 0.001) compared to control samples. However, compared to BPH, *miR‐483‐5p* and *miR‐574‐3p* were statistically significant (*p* < 0.001), and *miR‐141‐3p* and *miR‐375‐3p* were statistically significant (*p* < 0.05). Panel 2 consists of *miR‐21‐5p* and *miR‐148a‐3p*, which show significant differences in PCa compared to control samples but do not significantly distinguish PCa from BPH. All miRNAs in Panel 2 were statistically significant (*p* < 0.01) compared to control samples. Panel 3 consists of *miR‐21‐5p*, *miR‐141‐3p*, *miR‐148a‐3p*, *miR‐375‐3p*, *miR‐483‐5p*, and *miR‐574‐3p*, which show significant differences in BPH compared to control samples. In Panel 3, *miR‐21‐5p*, *miR‐375‐3p*, and *miR‐574‐3p* were statistically significant (*p* < 0.01), and *miR‐141‐3p*, *miR‐148a‐3p*, and *miR‐483‐5p* were statistically significant (*p* < 0.05) compared to control samples. As a result, the combination of Panels 1–3 not only can be used as biomarkers to diagnose PCa but also overcome the low sensitivity and specificity between PCa and BPH patients as a disadvantage of PSA testing.

## 3 DISCUSSION

High‐efficiency NAs sampling is required for noninvasive liquid biopsy applications such as early cancer detection and treatment prognosis monitoring. However, the conventional methods have disadvantages, such as time‐consuming, expensive, small sample processing capacity, and contamination by genomic NAs due to chaotropic and harmful reagents, besides the complexity of using equipment, such as large centrifuge, vacuum pump, and thermoregulator (Table 1). Our new platform, HAZIS‐CirR, allows simple and rapid enrichment and isolation of circRNAs using HHs to complement the existing methods. HHs, also known as carbonyl‐reactive crosslinkers, react with aldehydes of oxidized sugars to form stable conjugates through the hydrazone bond.^41^, ^42^, ^55^ HHs are commonly used for the preparation of specific glycoprotein conjugates and glycoprotein labeling and immobilization, and many studies have focused on protein interactions with HHs with regards to antibody labeling, metabolic labeling, circulating protein, and hydrazide‐modified nanomaterials, in addition to cancer‐related proteins for cancer diagnosis and therapy.^56^, ^57^, ^58^, ^59^, ^60^ However, despite several advantages of HHs, the applications for molecular diagnostic platforms have been insufficiently explored. We reported a sample preparation that combines AZ and syringe filtration for the first time to develop a novel molecular diagnostic platform utilizing the advantages of HHs. HAZIS‐CirR is inexpensive and does not use chaotropic reagents and lysis buffers that can damage NAs or increase the genomic background, and the entire process is completed within 20 min. In addition, it is low‐cost, simple, user‐friendly, and does not require electric equipment, such as a large centrifuge, vacuum pump, and thermoregulator (Table 1).

**TABLE 1:** Comparison of the HAZIS‐CirR with other RNA isolation methods

Types	HAZIS‐CirR	Spin‐column method^a^	Magnetic bead method^b^	Phenol/chloroform method^c^
Targets	Circulating NAs	Circulating NAs	Circulating NAs	miRNAs
Steps	Enrichment Isolation	Enrichment Isolation	Enrichment Isolation	Isolation
Volume capacity	1–10 ml >10 ml	1–5 ml	2 ml or 4 ml	0.2–1 ml
Toxicity	No	No	No	Yes (phenol/chloroform)
Electricity required	No	Yes	Yes	Yes
Special equipment	No	Vacuum pump Vacuum system Large centrifuge Thermoregulator	Magnet stand Large centrifuge Thermoregulator	Large centrifuge Thermoregulator
Lysis step	No	60°C for 30 min Ice for 5 min	65°C for 30 min Ice for 5 min	Ice for 10 min
Temperature	Room temperature	Room temperature Ice temperature 60°C 56°C	Room temperature Ice temperature 65°C 4°C	Room temperature Ice temperature 95°C
Cost per sample	$2	$25	$20	$10
Time	<20 min	>90 min	>90 min	>30 min

*Note*: Specifications and manufacturer's instructions of kit are referenced.

^a^ QIAamp Circulating Nucleic Acid Kit, catalog no. 55114, Qiagen.

^b^ MagMAX™ Cell‐Free Total Nucleic Acid Isolation Kit, catalog no. A36716, Thermo Fisher Scientific.

^c^ *mir*Vana™ miRNA Isolation Kit, with phenol, catalog no. AM1561, Thermo Fisher Scientific.

After HAZIS‐CirR optimization, we confirmed several results for diagnosing PCa via HAZIS‐CirR and demonstrated its clinical utility using urine samples. Enrichment and isolation of circRNA in HAZIS‐CirR has high capture efficiency using optimal AZ (5 mg ml^−1^) and ADH (50 mg ml^−1^) concentrations. In addition, there are no limitations on the volume capacity of the samples, the length of the RNAs, and the type of the liquid samples. To demonstrate the clinical utility of HAZIS‐CirR, we analyzed 89 clinical urine samples. The 100 μl of urinary circRNAs was concentrated and extracted using 4 ml of urine samples, followed by the analysis of the circulating mRNA and circulating miRNA for PCa diagnosis and differentiation from BPH and the control samples. Enrichment of circRNAs using such large amounts or entire samples can increase the sensitivity by eliminating the exclusion of circRNAs in the remaining samples that are not used in conventional methods due to the limitation of sample volume capacity. Circulating mRNA analysis suggested that *TMPRSS2‐ERG* showed a significant difference as a PCa diagnostic biomarker compared to BPH and the control samples. In the circulating miRNA analysis, *miR‐141‐3p*, *miR‐375‐3p*, *miR‐483‐5p*, and *miR‐574‐3p* showed significant differences as PCa diagnostic biomarkers compared to BPH and the control samples, and *miR‐21‐5p* and *miR‐148a‐3p* showed significant differences as PCa diagnostic biomarkers compared to the control. We identified and validated three miRNA panels; Panel 1 (PCa vs. BPH and the control groups), Panel 2 (PCa vs. the control group), and Panel 3 (BPH vs. the control group) that can differentiate among PCa, BPH, and control samples by comparing the miRNA levels. These results support previous studies that show tumor‐derived miRNAs enter the circulation system, and varying levels of circRNAs differ between patients with cancer and control patients and can be used as noninvasive biomarkers for cancer diagnosis.^3^, ^4^, ^5^, ^7^, ^22^, ^23^, ^24^ In addition, the three new miRNA panels consisting of six miRNAs may be used as potential circulating miRNA biomarkers for PCa diagnosis.

Despite these advantages of HAZIS‐CirR, there are three additional considerations to establish the clinical utility of circRNAs. (1) The exploration of circulating mRNAs for PCa diagnosis and further analysis of different types of circulating mRNAs are needed. Contrary to our expectation, circulating mRNA analysis using *PCA3* showed no significant difference among PCa, BPH, and the control samples, prompting a discussion on the use of urinary circulating mRNAs as PCa biomarkers. First, compared to circulating NAs in plasma, which are approximately 160–167 nts, urinary circulating NAs are fragmented through glomerular filtration, thus shorter in length (less than 100 nts) and more readily degraded. Oreskovic et al.^61^ suggested the development of an extraction platform for small‐sized fragmented NAs, necessity the selection of short‐length target sequences to use urinary circulating NAs in clinical settings. The extraction of NAs using conventional silica materials is not suitable for short‐length circulating NAs because it is a method dependent on the length of NAs due to hydrophobic interactions caused by dehydration of silica and NA surfaces and hydrogen bonding between silica and NA backbones.^62^, ^63^ This shows that HAZIS‐CirR may be more suitable as an enrichment and isolation platform for NAs capable of extracting small circulating mRNAs using covalent and electrostatic coupling. However, because primers and target sequences were designed under the same conditions used for cell‐derived mRNA, it is necessary to select short target sequences to use short circulating mRNA as a PCa diagnostic biomarker. The second is the low circulating mRNA extraction efficiency due to the absence of a lysis step. In the case of the existing extraction method for circulating NAs, a lysis step is performed to enhance the recovery rate of circulating NAs from a small sample volume.^7^, ^29^, ^64^, ^65^, ^66^ Although a lysis step can extract a high concentration of circulating NAs, contamination with chaotropic reagents of cell‐derived genomic NAs, and prostate‐related NAs extracted from PCa cells and urinary exosomes may reduce the sensitivity and specificity in analyzing pure circulating NAs. From this perspective, further analysis and exploration of pure circulating NAs in urine through the development of a circulating NA enrichment and isolation platform without lysis are essential. We anticipate that further analysis and expansion of HAZIS‐CirR in various clinical settings will complement the limitations of circulating mRNA analysis and further validate its clinical utility as a cancer biomarker.

(2) Circulating miRNA analysis using additional clinical samples and validation of miRNAs panels are required. We verified six miRNA biomarkers (*miR‐21‐5p*, *miR‐141‐3p*, *miR‐148a‐3p*, *miR‐375‐3p*, *miR‐483‐5p*, and *miR‐574‐3p*) and three miRNA panels by analyzing circulating miRNAs from 62 urine samples for PCa diagnosis and differentiation from BPH and control samples; however, this represents a relatively small clinical sample size. Numerous studies have focused on the development of molecular diagnostic technology for cancer diagnosis. As new technologies are developed, it is critical to standardize sample preparation and NA detection procedures, as well as clinical validation utilizing large‐scale clinical samples. Consequently, large‐scale and independent validation of HAZIS‐CirR as a sample preparation platform is needed. Nevertheless, we expect that HAZIS‐CirR will solve the limitations of low detection sensitivity of existing detection methods by obtaining a high concentration of miRNA through concentration and extraction of the low concentration of circulating miRNA present in patient samples and be used as a platform for early and precision cancer diagnosis.

(3) It is necessary to confirm the clinical usefulness of HAZIS‐CirR using various types of tumors and circRNAs, and in‐depth verification of circRNAs as noninvasive biomarkers through additional analysis and exploration is required. The application of liquid biopsy is a promising field for the noninvasive analysis of personalized biomolecules in real‐time, including circRNAs. An innovative platform will be instrumental for prognostic and predictive screening to establish treatment and impact early cancer diagnosis. circRNAs have been used as potential biomarkers, and their suitability as diagnostic, prognostic, and predictive biomarkers for specific cancer treatments has been proposed.^2^, ^22^, ^67^, ^68^, ^69^, ^70^, ^71^, ^72^

## 4 MATERIALS AND METHODS

### 4.1 Fabrication and operation of HAZIS‐CirR

Amine modification of zeolite (96096‐100G; Sigma‐Aldrich, St. Louis, MO, USA) was performed via silanization in several steps. First, zeolite (3 g) was washed in ultrapure distilled water (DW; 150 ml) and 95% ethanol‐DW (95:5, v/v) by stirring at 550 rpm for 10 min at room temperature (RT). For silanization, the washed zeolite was stirred in 2% APDMS (371890‐50ML, Sigma‐Aldrich)‐95% ethanol (2:98, v/v; 150 ml) at 450 rpm for 4 h at RT. After silanization, AZ was washed several times with ultrapure DW and 95% ethanol (150 ml) by stirring at 550 rpm for 10 min at RT. Afterward, AZ was completely dried using a vacuum chamber and pump and stored at RT until use.

The enrichment and isolation of circRNAs by HAZIS‐CirR involved four steps: (1) sample mixing and incubation, (2) enrichment, (3) washing, and (4) isolation. Briefly, a solution (1 ml) containing *hsa‐mir‐21‐5p* mimics (Shanghai GenePharma Co., Ltd., Shanghai, China) was used for the optimization of HAZIS‐CirR. Urine samples (4 ml) were analyzed for circRNAs via HAZIS‐CirR to confirm the clinical utility of the platform. (1) In the sample mixing and incubation step, AZ (2.5, 5.0, or 10 mg) and ADH (10, 25, or 50 mg; 8.41689.0050, Merck Millipore), one of the HHs, were added to the sample (per 1 ml). ADH was selected by optimization of HAZIS‐CirR using HHs: CDH (C11006‐100G; Sigma‐Aldrich), ODH (131296‐25G; Sigma‐Aldrich), MDH (M3206; Tokyo Chemical Industry, Tokyo, Japan), and SDH (S5502‐25G; Sigma‐Aldrich). After sample mixing, incubation for covalent and electrostatic reactions was performed for 10 min at RT. (2) In the enrichment step, the incubated samples (1–4 ml) were passed through a sterile 0.45‐μm PVDF syringe filter (FJ13BSCPV004AL01; GVS Filter Technology, Indianapolis, IN, USA) using a syringe (1.5 ml volume capacity; Korea Vaccine Co., Ltd., Yongin, Korea). circRNAs captured with AZ by covalent and electrostatic coupling were filtered based on the size of AZ, and debris not captured with AZ passed through the filter and was discarded. (3) Remaining contaminants were removed by washing with PBS (3 ml). (4) In the isolation step, sodium bicarbonate (100 μl, 50 mM; S5761‐500G, Sigma‐Aldrich) elution buffer (pH 10.6) was used to hydrolyze the crosslinks between circRNAs and ADH on the AZ. Eluted circRNAs were collected in microcentrifuge tubes (1.5 ml volume capacity) and stored at sub‐zero temperatures (−20 or −80°C) until further use.

### 4.2 Evaluation of HAZIS‐CirR

To evaluate the detection limit and capture efficiency of circRNA in HAZIS‐CirR, *hsa‐mir‐21‐5p* ss mimic (from 1.204 × 10^13^ copies reaction^−1^, 20 μM to 1.204 × 10^0^ copies reaction^−1^, 2 aM) and T7 in vitro transcribed RNAs of 150 bp (from 1.14 × 10^10^ copies reaction^−1^ to 1.14 × 10^4^ copies reaction^−1^) and 112 bp (from 1.35 × 10^10^ copies reaction^−1^ to 1.35 × 10^4^ copies reaction^−1^) lengths were prepared by serial dilutions in 1.5 ml microcentrifuge tubes or 15 ml polypropylene tubes. The serial diluted samples were stored at sub‐zero temperatures (−20 or −80°C) until use. The zeta‐potential, FTIR, and SEM images of zeolite, AZ, HAZ, and HAZ‐NAs were acquired using a DLS device (Nano ZS DLS, Malvern Panalytical Ltd., UK), FTIR spectrometer (Vertex 70; Bruker, Germany), and field‐emission SEM (JSM‐7800F Prime; JEOL Ltd., Japan), respectively.

### 4.3 Conventional methods

circRNAs were reverse‐transcribed into cDNA using the QuantiTect Reverse Transcription Kit (205313; Qiagen, Hilden, Germany) for mRNA, and the Mir‐X miRNA First‐Strand Synthesis Kit (638313; Takara Bio USA, Inc., Kyoto, Japan) for miRNA, according to the respective manufacturer's instructions. The synthesized cDNAs were stored at −20°C until use. For the synthesis of long RNA fragments, PCR products containing *PCA3* and 18S rRNA target genes were obtained using QIAGEN OneStep RT‐PCR Kit (210212; Qiagen, Hilden, Germany). The purified T7 in vitro transcribed RNAs of 150 bp and 112 bp containing *PCA3* and 18S rRNA target genes were synthesized using MEGAscript T7 Transcription Kit (AMB13345; Ambion, Thermo Fisher Scientific, Waltham, MA, USA) and MEGAclear Transcription Clean‐Up Kit (AM1908; Invitrogen, Thermo Fisher Scientific). The concentration of synthesized T7 in vitro transcribed RNA was measured using a NanoDrop One/OneC spectrophotometer (Thermo Fisher Scientific). For use of human serum, sterile filtered heat‐inactivated human male AB plasma was used (H3667; Sigma‐Aldrich). For circulating mRNA analysis, SYBR Green Realtime PCR master mix (QPK‐201, Toyobo, Osaka, Japan) was used for detection of *PCA3*, *TMPRSS2‐ERG*, and 18S rRNA. The forward and reverse primers were synthesized at the usual length of around 24 bp (Table S7). The amplification protocol consisted of an initial denaturation step at 95°C for 20 s, 40 cycles of 3 s at 95°C, and 30 s at 60°C, and the melt curve stage consisted of 95°C for 15 s, a melt from 60°C for 1 min to 95°C for 15 s with a ramp rate of 1%, followed by 60°C for 15 s. The amplified products with SYBR Green signals were obtained using an Applied Biosystems™ 7500 Real‐Time PCR System (Applied Biosystems, MA, USA). For circulating miRNA analysis, the TB Green Advantage qPCR Premix Kit (639676; Takara Bio USA, Inc.) was used for detection of *hsa‐miR‐21‐5p*, *hsa‐miR‐141‐3p*, *hsa‐miR‐375‐3p*, *hsa‐miR‐148a‐3p*, *hsa‐miR‐483‐5p*, *hsa‐miR‐574‐3p*, and the noncoding small nuclear RNA component of U6 snRNP (U6 snRNA). The mature sequences were used as forward primers, and the miRNA reverse primers of miRNA and U6 snRNA primers were provided in the kit (Table S1). The amplification protocol consisted of an initial denaturation step at 95°C for 10 s, 40 cycles of 5 s at 95°C, 20 s at 60°C, and a cooling step at 40°C for 30 s. The amplified products with TB Green signals were obtained using a QuantStudio 3 Real‐Time PCR System (Thermo Fisher Scientific). To separate the PCR products, gel electrophoresis was performed on a 2% agarose gel containing LoadingSTAR (A750, Dyne Bio, Inc., Seoul, Korea). The gel was visualized using a ChemiDoc XRS+ system (Bio‐Rad, Marnes‐la‐Coquette, France).

### 4.4 Analysis of circRNAs

The RQ value of circRNAs was calculated using the comparative *C*_*t*_ method with 18S rRNA for mRNA and U6 snRNA for miRNA as the respective reference gene. The RQ value of gene expression was computed using Equations ((1), (2), (3)):

(1) $$\Delta C_{t}=C_{t}\;\left(\text{Target gene}\right)-C_{t}\;\left(\text{Reference gene}\right)$$

(2) $$\Delta \Delta C_{t}=\Delta C_{t}\;\left(\mathrm{PCa},\mathrm{BPH},\text{or control}\right)-\Delta C_{t}\;\left(\text{Average of control}\right)$$

(3) $$\text{Relative Quantification}\;\left(\mathrm{RQ}\right)\;\text{value}=2^{-\Delta \Delta \mathrm{Ct}}$$

The *p* values were determined by the unpaired *t*‐test, and graphs were obtained using GraphPad Prism 8 statistical software (GraphPad Software, San Diego, CA, USA).

### 4.5 Clinical specimens

For clinical analysis, urine samples were collected from 55 patients with PCa, 24 patients with BPH, and 10 control patients at the Asan Medical Center (AMC), Seoul, South Korea (Figure S6 and Table S8). All subjects gave their informed consent for inclusion before they participated in the study. The study was conducted in accordance with the Declaration of Helsinki, and the protocol was approved by the Ethics Committee of AMC. The use of patient information in this study was approved by the AMC (IRB no. 2019‐1312). Urine samples were selected from patients of similar age. The PSA levels of patients with PCa were relatively higher than those of the other groups (Table S8).

## 5 CONCLUSION

HAZIS‐CirR platform enables to concentrate rare circRNAs using large sample volumes, allowing extraction of high‐concentration circRNAs within 20 min in prostate cancers. HAZIS‐CirR does not use chaotropic and hazardous reagents because there is no cell lysis process, and it does not require electricity‐consuming equipment, such as centrifuge and thermoregulator. We identified three PCa miRNAs panels and one circulating mRNA using HAZIS‐CirR but only focused on diagnosis. This is a limited study and does not reflect the various functions of circRNAs. In addition, further studies are needed to fundamentally investigate the role of circRNAs and explore potential biological functional mechanisms, such as intercellular communication. However, the potential of circRNAs as cancer biomarkers to overcome human health problems is attractive and cannot be underestimated. In this respect, the development of molecular diagnostic platforms utilizing nanotechnology, such as HAZIS‐CirR, will help create more accurate and stable diagnostic and treatment approaches. We envision that HAZIS‐CirR will provide a new dimension for overcoming cancer by concentrating and extracting circulating NAs in various clinical fields not only for early and accurate diagnosis of cancer but also for metastasis confirmation and treatment prognosis.

## AUTHOR CONTRIBUTIONS

**Bonhan Koo:** Data curation (equal); formal analysis (equal); methodology (equal); resources (equal); validation (equal); writing – original draft (equal). **Yunlim Kim:** Investigation (equal); validation (equal). **Yoon Ok Jang:** Formal analysis (equal); validation (equal). **Huifang Liu:** Investigation (equal); validation (equal). **Myoung Gyu Kim:** Methodology (equal); validation (equal). **Hyo Joo Lee:** Methodology (equal); validation (equal). **Myung Kyun Woo:** Investigation (equal); validation (equal). **Yong Shin:** Conceptualization (equal); funding acquisition (equal); investigation (equal); project administration (equal); supervision (equal); writing – original draft (equal); writing – review and editing (equal).

## CONFLICT OF INTERESTS

The authors declare no competing interests.

### PEER REVIEW

The peer review history for this article is available at https://publons.com/publon/10.1002/btm2.10348.

## Supporting information


**Appendix S1** Supporting Information

## Data Availability

The data that support the findings of this study are available from the corresponding author upon reasonable request.
