# Supplementary material for: A novel platform using homobifunctional hydrazide for enrichment and isolation of urinary circulating RNAs
Source: Bioeng Transl Med. 2022 Jun 3;8(1):e10348. doi: 10.1002/btm2.10348 (PMC9842063; doi:10.1002/btm2.10348)
Supplement: Supplementary file 1 — Appendix S1 Supporting Information [file BTM2-8-e10348-s001.docx]

Supplementary Information

A novel platform using homobifunctional hydrazide for enrichment and isolation of urinary circulating RNAs

Bonhan Koo^1^, Yunlim Kim^2^, Yoon Ok Jang^1^, Huifang Liu^1^, Myoung Gyu Kim^1^, Hyo Joo Lee^1^, Myung Kyun Woo^3^, Choung-Soo Kim^2,4*^, Yong Shin^1,*^

^1^Department of Biotechnology, College of Life Science and Biotechnology, Yonsei University, 50 Yonsei-ro, Seodaemun-gu, Seoul 03722, Republic of Korea

^2^Department of Urology, Asan Medical Center, University of Ulsan College of Medicine, 88 Olympic-ro 43-gil, Songpa-gu, Seoul 05505, Republic of Korea

^3^Department of Electrical Engineering, University of Ulsan, 93 Daehak-ro, Nam-gu, Ulsan, Republic of Korea

^4^Department of Urology, Ewha Womans University Mokdong Hospital, 1071 Anyangcheon-ro, Yangcheon-gu, Seoul 07985, Republic of Korea

* Correspondence and request for materials should be addressed to Y. Shin (shinyongno1@yonsei.ac.kr) and C.-S. Kim (cskim@eumc.ac.kr)


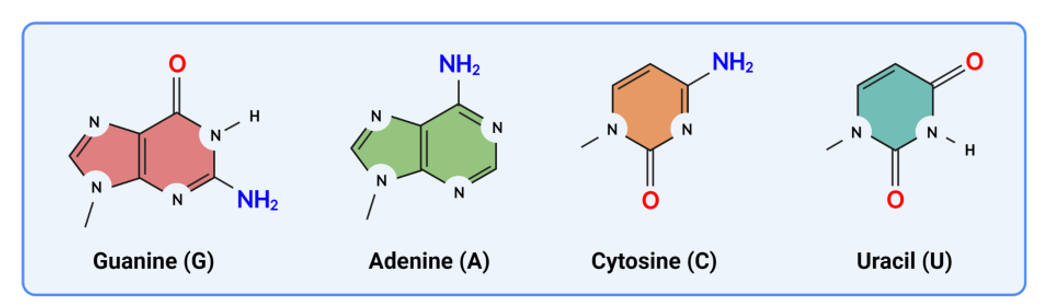


**Figure S1. Functional groups of nitrogenous bases of RNA in HAZIS-CirR.** This image was created using BioRender (https://biorender.com).

**
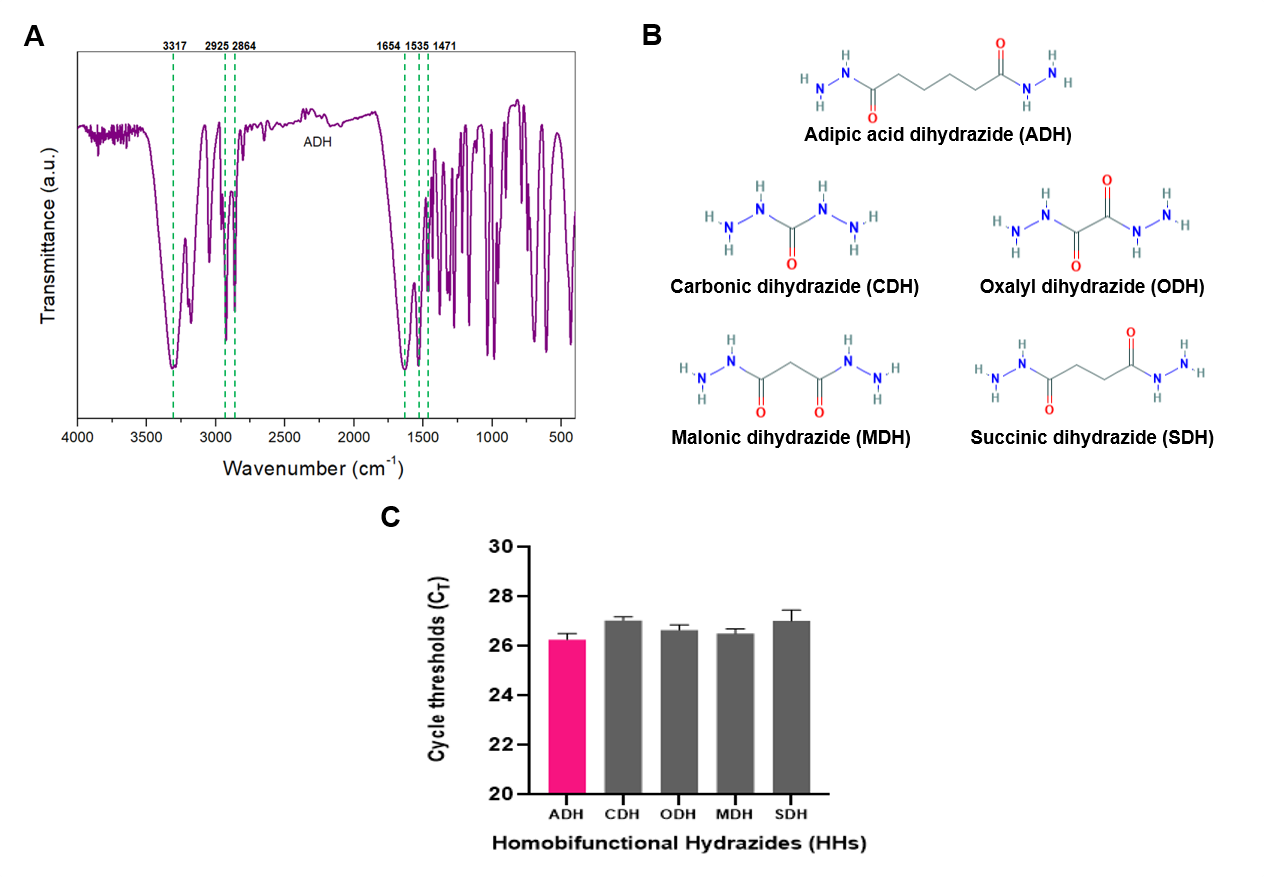
**

**Figure S2. Characterization of homobifunctional hydrazides (HHs). (A)** Fourier-transform infrared spectroscopy (FTIR) analysis of adipic acid dihydrazide (ADH). **(B)** Structures of homobifunctional hydrazides: adipic dihydrazide (ADH), carbonic dihydrazide (CDH), oxalyl dihydrazide (ODH), malonic dihydrazide (MDH), and succinic dihydrazide (SDH). **(C)** Circulating RNA capture efficiency of HAZIS-CirR using HHs. Error bars indicate standard deviation of the mean of at least three independent experiments.

**
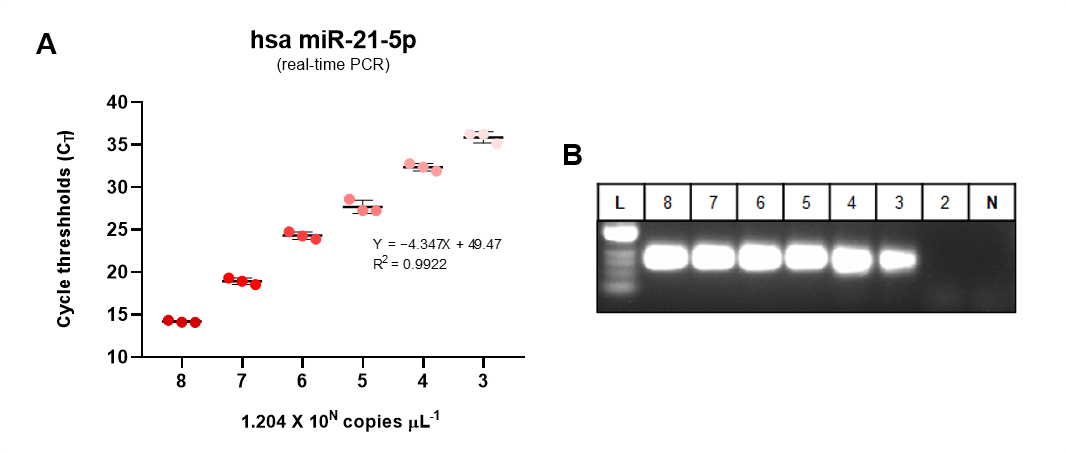
**

**Figure S3.** **Capacity of real-time PCR with the *hsa-miR-21-5p* ss mimics.** **(A)** Linear relationship and detection limit of real-time PCR assay using *hsa-miR-21-5p* ss mimics serially diluted from 1.204×10^8^ to 1.204×10^2^ copies µL^−1^. **(B)** Gel electrophoresis data for the detection of *hsa-miR-21-5p* ss mimics. (L: 50-bp DNA ladder, 8-2: 1.204×10^N^ copies µL^−1^, N: negative control)


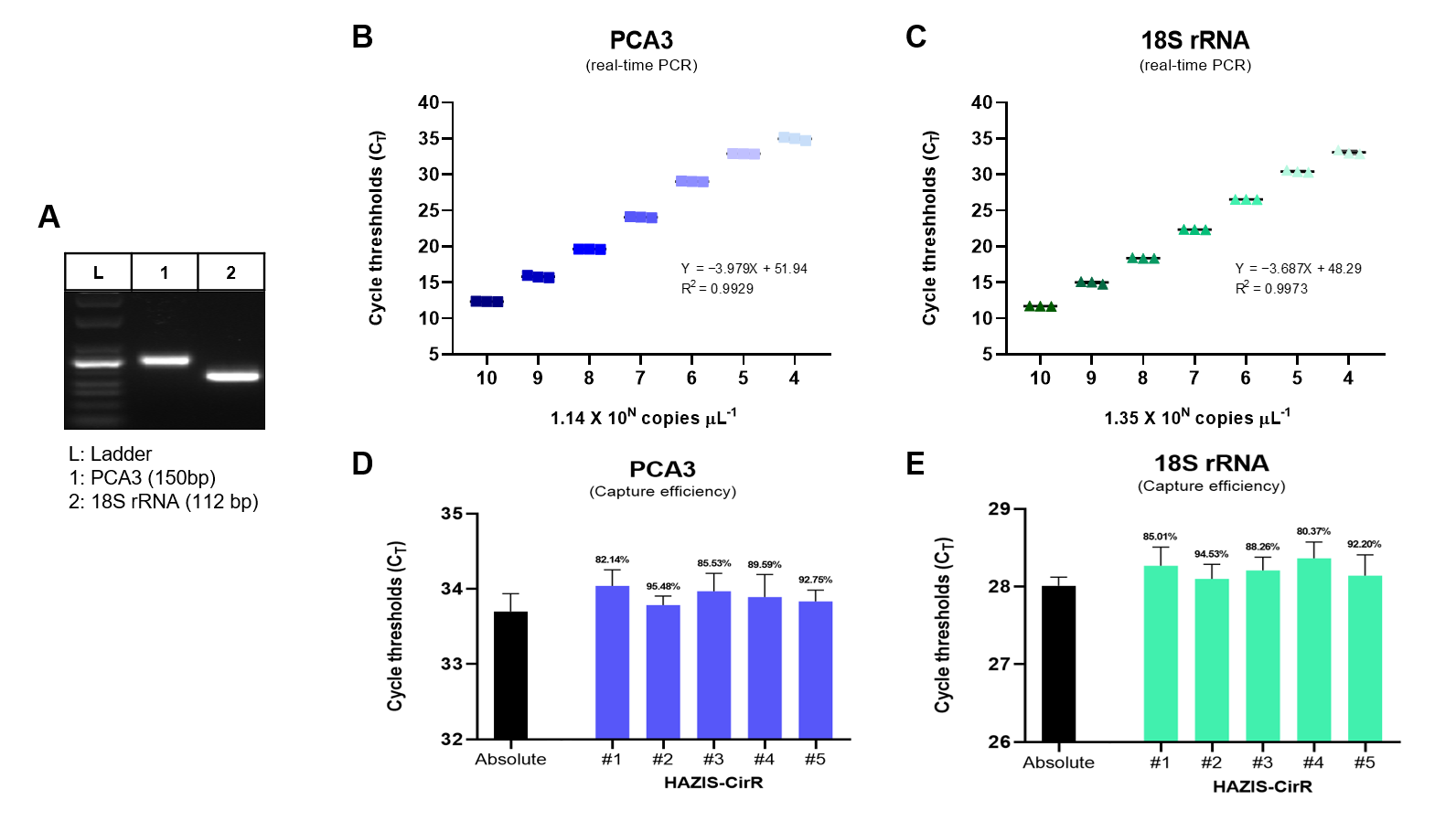


**Figure S4.** **Capture efficiency using** **T7 in vitro transcribed RNAs for long RNA fragments enrichment and isolation in HAZIS-CirR**. **(A)** Gel electrophoresis data for the detection of T7 in vitro transcribed RNAs of 150 bp containing PCA3 and 112 bp containing 18S rRNA target genes. **(B−C)** Linear relationship of real-time PCR assay using 150 bp and 112 bp lengths of T7 in vitro transcribed RNAs serially diluted from 1.14×10^10^ to 1.14×10^4^ copies µL^−1^ and 1.35×10^10^ to 1.35×10^4^ copies µL^−1^, respectively. **(D−E)** Capture efficiency of HAZIS-CirR according to lengths of circulating RNAs. Capture efficiency was calculated by quantitative comparison using the linear relationship of the standard curve.


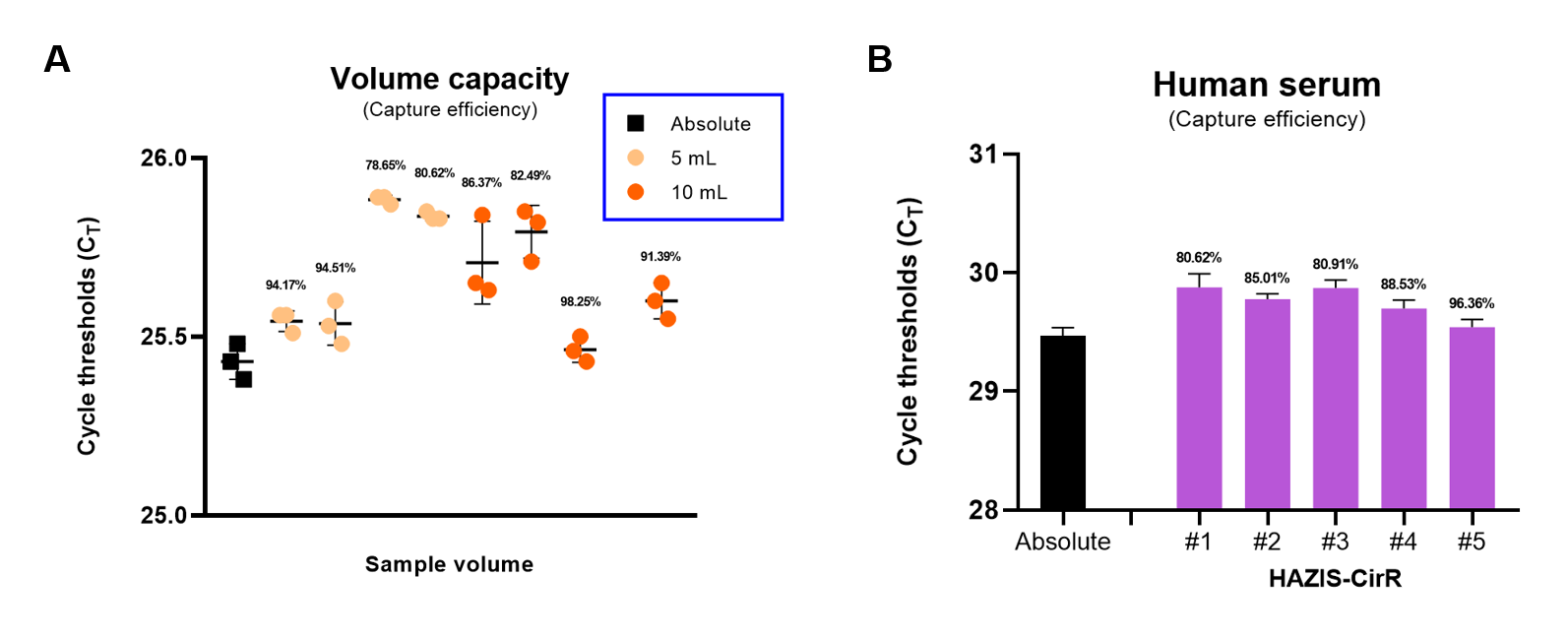


**Figure S5.** **Capture efficiency using 5 mL and 10 mL volume of samples and human serum in HAZIS-CirR**. **(A)** Capture efficiency of HAZIS-CirR using 5 mL and 10 mL volume of samples. **(B)** Capture efficiency of HAZIS-CirR using human serum. Capture efficiency was calculated by quantitative comparison using the linear relationship of the standard curve.


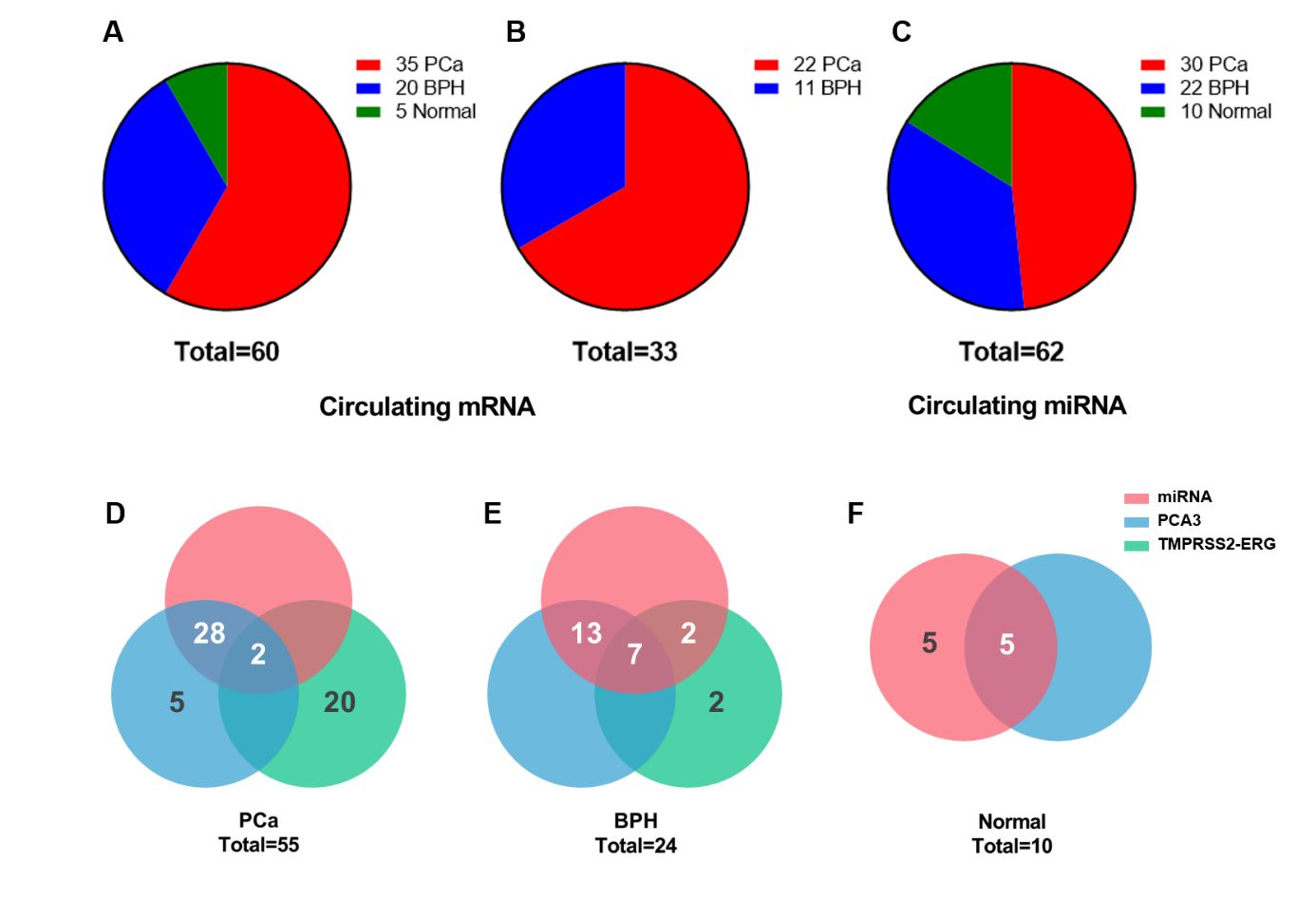


**Figure S6.** **Pie chart showing the distribution of clinical samples in HAZIS-CirR.** Distribution for **(A−B)** circulating mRNA and **(C)** circulating miRNA analysis. Distribution for **(D)** PCa, **(E)** BPH, and **(F)** controls. PCa, prostate cancer; BPH, benign prostatic hyperplasia.

**Table S1. Raw data of standard curve using *hsa-mir-21-5p* ss mimics**

| **Replicate No.** | **1.204 × 10^N^ copies reaction^−1^**  (C_T_) | | | | | |
| --- | --- | --- | --- | --- | --- | --- |
|  | **8** | **7** | **6** | **5** | **4** | **3** |
| #1 | 14.121 | 18.533 | 23.883 | 27.251 | 31.902 | 35.100 |
| #2 | 14.333 | 18.933 | 24.256 | 27.249 | 32.396 | 36.272 |
| #3 | 14.093 | 19.323 | 24.770 | 28.600 | 32.791 | 36.241 |
| **Mean** | **14.182** | **18.930** | **24.303** | **27.700** | **32.363** | **35.871** |
| **STDEV** | **0.132** | **0.395** | **0.445** | **0.779** | **0.446** | **0.668** |

**Table S2. Raw data of standard curve using T7 in vitro transcribed RNAs of 150 bp containing PCA3 and 112 bp containing 18S rRNA target gene**

| **Replicate No.** | **150 bp containing PCA3 gene**  (1.14 × 10^N^ copies reaction^−1^, C^T^) | | | | | | **112 bp containing 18S rRNA gene**  (1.35 × 10^N^ copies reaction^−1^, C^T^) | | | | |
| --- | --- | --- | --- | --- | --- | --- | --- | --- | --- | --- | --- |
|  | **#1** | **#2** | **#3** | **Mean** | **STDEV** | **#1** | | **#2** | **#3** | **Mean** | **STDEV** |
| **10** | 12.33 | 12.36 | 12.31 | **12.333** | **0.025** | 11.65 | | 11.68 | 11.71 | **11.680** | **0.030** |
| **9** | 15.94 | 15.67 | 15.75 | **15.787** | **0.139** | 15.12 | | 14.77 | 15.04 | **14.977** | **0.183** |
| **8** | 19.62 | 19.61 | 19.63 | **19.620** | **0.010** | 18.35 | | 18.42 | 18.33 | **18.367** | **0.047** |
| **7** | 23.98 | 24.11 | 24.07 | **24.053** | **0.067** | 22.31 | | 22.37 | 22.35 | **22.343** | **0.031** |
| **6** | 28.98 | 29.08 | 29.01 | **29.023** | **0.051** | 26.51 | | 26.54 | 26.55 | **26.533** | **0.021** |
| **5** | 32.89 | 32.84 | 32.86 | **32.863** | **0.025** | 30.58 | | 30.29 | 30.38 | **30.417** | **0.148** |
| **4** | 34.72 | 35.15 | 34.99 | **34.953** | **0.217** | 32.86 | | 33.39 | 32.97 | **33.073** | **0.280** |

**Table S3. Raw data of capture efficiency determined using *hsa-mir-21-5p* ss mimics in HAZIS-CirR**

| **Replicate No.** | **1.204 × 10^N^ copies reaction^−1^**  (C_T_) | | | | |
| --- | --- | --- | --- | --- | --- |
|  | **7** | **6** | **5** | **4** | **3** |
| #1 | 19.173 | 24.513 | 27.513 | 31.984 | 35.984 |
| #2 | 19.057 | 24.615 | 27.615 | 32.849 | 36.349 |
| #3 | 19.365 | 24.487 | 28.487 | 32.707 | 36.407 |
| **Mean** | **19.198** | **24.539** | **27.872** | **32.514** | **36.247** |
| **Capture efficiency (%)** | **86.81** | **88.34** | **91.35** | **92.38** | **82.03** |

**Table S4. Raw data of capture efficiency determined using T7 in vitro transcribed RNAs of 150 bp containing PCA3 and 112 bp containing 18S rRNA target gene in HAZIS-CirR**

| **Replicate No.** | **150 bp containing PCA3 gene**  (C^T^) | | | | | | **112 bp containing 18S rRNA gene**  (C^T^) | | | | |
| --- | --- | --- | --- | --- | --- | --- | --- | --- | --- | --- | --- |
|  | **#1** | **#2** | **#3** | **Mean** | **Capture**  **efficiency (%)** | **#1** | | **#2** | **#3** | **Mean** | **Capture**  **efficiency (%)** |
| **Test #1** | 33.97 | 34.28 | 33.87 | **34.04** | **82.14** | 28.18 | | 28.54 | 28.09 | **28.27** | **85.01** |
| **Test #2** | 33.73 | 33.92 | 33.70 | **33.78** | **95.48** | 27.89 | | 28.25 | 28.16 | **28.10** | **94.53** |
| **Test #3** | 34.19 | 34.00 | 33.72 | **33.97** | **85.53** | 28.42 | | 28.54 | 28.13 | **28.36** | **80.37** |
| **Test #4** | 33.56 | 33.96 | 34.15 | **33.89** | **89.59** | 28.40 | | 28.15 | 28.08 | **28.21** | **88.26** |
| **Test #5** | 33.68 | 33.84 | 33.98 | **33.83** | **92.75** | 27.83 | | 28.30 | 28.29 | **28.14** | **92.20** |

**Table S5. Raw data of capture efficiency determined using 5 mL and 10 mL containing *hsa-mir-21-5p* ss mimic in HAZIS-CirR**

| **Replicate No.** | **5 mL**  (C^T^) | | | | | | **10 mL**  (C^T^) | | | | |
| --- | --- | --- | --- | --- | --- | --- | --- | --- | --- | --- | --- |
|  | **#1** | **#2** | **#3** | **Mean** | **Capture**  **efficiency (%)** | **#1** | | **#2** | **#3** | **Mean** | **Capture**  **efficiency (%)** |
| **Test #1** | 25.56 | 25.51 | 25.56 | **25.54** | **94.17** | 25.84 | | 25.65 | 25.63 | **25.71** | **86.37** |
| **Test #2** | 25.60 | 25.48 | 25.53 | **25.54** | **94.51** | 25.85 | | 25.82 | 25.71 | **25.79** | **82.49** |
| **Test #3** | 25.87 | 25.89 | 25.89 | **25.88** | **78.65** | 25.43 | | 25.46 | 25.50 | **25.46** | **98.25** |
| **Test #4** | 25.83 | 25.85 | 25.83 | **25.84** | **80.62** | 25.65 | | 25.60 | 25.55 | **25.60** | **91.39** |

**Table S6. Raw data of capture efficiency determined using human serum containing *hsa-mir-21-5p* ss mimics in HAZIS-CirR**

| **Replicate No.** | **Human serum**  (C_T_) | | | | |
| --- | --- | --- | --- | --- | --- |
|  | **Test #1** | **Test #2** | **Test #3** | **Test #4** | **Test #5** |
| #1 | 29.91 | 29.73 | 29.83 | 29.62 | 29.55 |
| #2 | 29.97 | 29.78 | 29.95 | 29.73 | 29.60 |
| #3 | 29.75 | 29.82 | 29.83 | 29.75 | 29.47 |
| **Mean** | **29.88** | **29.78** | **29.87** | **29.70** | **29.54** |
| **Capture efficiency (%)** | **80.62** | **85.01** | **80.91** | **88.53** | **96.36** |

**Table S7. Primer sequences used in this study**

| **Target** | | **Location** | **Sequence (5'–3')** | **Length**  (bp) |
| --- | --- | --- | --- | --- |
| **mRNA** | **PCA3** | Forward | GAG AAC AGG GGA GGG AGA G | 19 |
|  |  | Reverse | ACG TTC TGG GAT ACA TGT GC | 20 |
|  | **TMPRSS2-**  **ERG fusion** | Forward | CCT GGA GCG CGG CAG GAA GCC TTA TCA GTT G | 31 |
|  |  | Reverse | TCC TGC TGA GGG ACG CGT GGG CTC ATC TTG | 30 |
|  | **18S rRNA** | Forward | CCT GGA TAC CGC AGC TAG GA | 20 |
|  |  | Reverse | GCG GCG CAA TAC GAA TGC CCC | 21 |
| **microRNA** | ***hsa-miR-21-5p*** | Forward | TAG CTT ATC AGA CTG ATG TTG A | 22 |
|  | ***hsa-miR-141-3p*** | Forward | TAA CAC TGT CTG GTA AAG ATG G | 22 |
|  | ***hsa-miR-375-3p*** | Forward | TTT GTT CGT TCG GCT CGC GTG A | 22 |
|  | ***hsa-miR-148a-3p*** | Forward | TCA GTG CAC TAC AGA AGT TTG T | 22 |
|  | ***hsa-miR-483-5p*** | Forward | AAG ACG GGA GGA AAG AAG GGA G | 22 |
|  | ***hsa-miR-574-3p*** | Forward | CAC GCT CAT GCA CAC ACC CAC A | 22 |
|  | **Universal** | Reverse | mRQ 3' Primer (provided) | . |
|  | **U6 snRNA** | Forward | U6 Forward Primer (provided) | . |
|  |  | Reverse | U6 Reverse Primer (provided) | . |

**Table S8. Demographic characteristics for circulating RNAs analysis in HAZIS-CirR**

| Demographics in HAZIS-CirR | | Age (median, IQR) | Initial PSA value |
| --- | --- | --- | --- |
| Circulating mRNA  (PCA3) | PCa (35) | 70 (63-72) | 6.60 (4.00-12.69) |
|  | BPH (20) | 68 (63.3-74) | 4.62 (3.35-7.09) |
|  | Normal (5) | 64 (60-68.8) | 2.31 (1.50-6.29) |
| Circulating mRNA  (TMPRSS2-ERG gene fusion) | PCa (22) | 70 (68-72.8) | 5.06 (3.70-8.93) |
|  | BPH (11) | 70 (63.5-75) | 3.13 (1.64-4.70) |
| Circulating miRNAs | PCa (30) | 70 (62.5-73) | 6.56 (4.30-12.09) |
|  | BPH (22) | 68 (62.5-74) | 4.45 (3.13-6.61) |
|  | Normal (10) | 68.0 (66-71) | 2.31 (0.86-6.98) |
